# Cell Differentiation of Pluripotent Tissue Sheets Immobilized on Supported Membranes Displaying Cadherin-11

**DOI:** 10.1371/journal.pone.0054749

**Published:** 2013-02-12

**Authors:** Alexander Körner, Christina Deichmann, Fernanda F. Rossetti, Almut Köhler, Oleg V. Konovalov, Doris Wedlich, Motomu Tanaka

**Affiliations:** 1 Physical Chemistry of Biosystems, Physical Chemistry Institute, University of Heidelberg, Heidelberg, Germany; 2 Cell and Developmental Biology, Zoological Institute, Karlsruhe Institute of Technology (KIT), Karlsruhe, Germany; 3 European Synchrotron Radiation Facility (ESRF), Grenoble, France; 4 Cell Biophysics Laboratory, Institute of Toxicology and Genetics, Karlsruhe Institute of Technology (KIT), Karlsruhe, Germany; University of California, San Diego, United States of America

## Abstract

Investigating cohesive tissue sheets in controlled cultures still poses a challenge since the complex intercellular interactions are difficult to mimic in *in vitro* models. We used supported lipid membranes functionalized by the adhesive part of the extracellular domain of the cell adhesion molecule cadherin-11 for the immobilization of pluripotent tissue sheets, the animal cap isolated from *Xenopus laevis* blastula stage embryos. Cadherin-11 was bound via histidine tag to lipid membranes with chelator head groups. In the first step, quantitative functionalization of the membranes with cadherin-11 was confirmed by quartz crystal microbalance and high energy specular X-ray reflectivity. In the next step, animal cap tissue sheets induced to neural crest cell fate were cultured on the membranes functionalized with cadherin-11. The adhesion of cells within the cohesive tissue was significantly dependent on changes in lateral densities of cadherin-11. The formation of filopodia and lamellipodia in the cohesive tissue verified the viability and sustainability of the culture over several hours. The expression of the transcription factor *slug* in externally induced tissue demonstrated the applicability of lipid membranes displaying adhesive molecules for controlled differentiation of cohesive pluripotent tissue sheets.

## Introduction

Biological membranes are key components of all living systems, forming the outer boundary of living cells or of internal cell compartments (organelles). They consist largely of a lipid bilayer that imparts a fluid character. Since 1980’s, planar lipid membrane models on solid substrates (called supported membranes) are widely used as biological membrane models that can be subjected to various surface sensitive techniques [1]–[4].

Supported membranes can readily be functionalized either by spreading vesicles incorporating transmembrane proteins (proteoliposomes) or by incorporating ‘anchor’ molecules for engineered proteins. This method is a powerful tool for creating complex experimental cell-surface models that can be investigated in a quantitative manner [1], [5]–[9]. One of the commonly used strategies is to incorporate proteins functionalized with glycerophosphatidylinositol (GPI) anchors [10], utilizing native anchors for many membrane proteins. As synthetic analogues to GPI anchors, lipid anchors with biotin head groups [11], [12] and those with nitrilotriacetic acid (NTA) head groups [13], [14] have been also used to couple various recombinant proteins and carbohydrates with biotin and histidine tags to the membrane surfaces, respectively.

Supported membranes displaying various functions can be used as the quantitative model of surrogate cells to understand the basic principles of various important cellular processes, such as the growth of adhesion patches according to the coalescence of specific pairs by the combination of experiments [5], [10], [15] and theoretical modeling [16]–[18]. An increasing number of studies suggests that coordinated cell movements, such as sorting and migration, are guided through the physical contacts between cells and tissues, which in turn are governed by tension and specific adhesion [19]–[24]. However, despite of a number of recent reports dealing with single cells on supported membranes, there have been no systematic studies that utilize supported membranes to guide cell differentiation by the immobilization of explanted tissues.

The primary aim of this work is (a) to realize the immobilization of pluripotent tissue sheets on supported lipid membranes without disrupting their connective structures and (b) to utilize such two-dimensional interfaces for the regulation of cell differentiation, which is a key process in development. Here, we first focus on the immobilization of animal caps explanted from *Xenopus laevis* embryos on functionalized supported membranes. The animal cap is isolated from *Xenopus* blastula stage embryos and represents a cohesive cell sheet of pluripotent cells, which are the equivalent to mammalian embryonic stem cells [25]. To date, the embryos of South African claw frog, *Xenopus laevis*, have been widely used to study vertebrate development due to their amenability to experimental manipulations and cell biological analysis. Neural crest cell (NCC) differentiation is a major process in the development of vertebrates, which occurs in response to bone morphogenetic protein (BMP), Wnt, retinoic acid (RA) and fibroblast growth factor (FGF) signaling in the embryonic ectoderm [26]–[29]. The NCCs are assumed to undergo an epithelial-to-mesenchymal transition (EMT) and leave the neuroectodermal tissue [30]. This process shares common features with the invasion and metastasis of cancer cells [31]–[33]. EMT involves the transcription factor *slug* that controls the transformation of non-motile epithelial cells into migrating cells. Therefore, a *slug* promoter reporter construct fused to GFP can be used as read-out system for a successful neural crest induction [34], [35].

**Figure 1:**
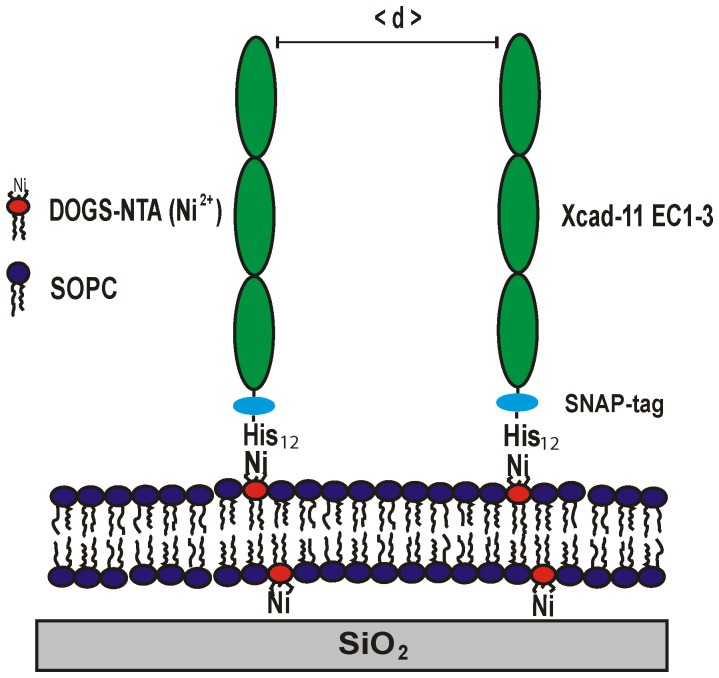
Supported membrane functionalized with Xcad-11 EC1-3 SNAP His12. As lipid anchors (DOGS-NTA) are uniformly mixed in matrix lipids (SOPC), the average distance between proteins <*d*> can be controlled within nm accuracy simply by adjusting the molar fraction of DOGS-NTA.

In this study, we functionalized the supported membranes with the adhesive part (EC1-3) of the recombinant protein *Xenopus* cadherin-11 (Xcad-11) fused to a histidine tag (Fig. 1), since previous accounts evidenced that Xcad-11 is crucial for neural crest formation [36] and that its extracellular domains mediate cell adhesion and migration of NCCs [36], [37]. As the first step, the binding of recombinant Xcad-11 with the histidine tag to lipid anchors carrying NTA head groups was monitored by quartz crystal microbalance with dissipation (QCM-D) technique [38], [39], confirming that the amount of proteins on the membrane can be controlled precisely by the molar fraction of NTA lipid anchors. The thickness, roughness, and electron density of supported membranes in the presence and absence of Xcad-11 molecules were determined by high energy specular X-ray reflectivity [40], [41]. After the quantitative characterization of the functionalized supported membranes, we placed *Xenopus* animal caps onto supported membranes displaying Xcad-11 at various average distances <*d*> and monitored the adhesion of animal cap cells *in vivo* using reflection interference contrast microscopy (RICM). After the optimization of the lateral density of Xcad-11, the animal caps were cultivated on the supported membranes. To induce the formation of NCC in the animal caps, we injected truncated bone morphogenetic protein receptor (tBR) and *Xenopus* Frizzled 7 (XFz7) as mRNA into one blastomere of a *Xenopus laevis* embryo at the two-cell stage. Animal caps were explanted from the blastocoel roof at the early blastula stage and cultured on the functionalized lipid membranes. After exploring the optimal conditions for maintaining the animal caps viable on functionalized supported membranes, we co-injected an additional reporter (*slug*-promoter-GFP) to gain experimental evidence of neural crest induction on supported membranes. The details of the experimental findings are described in the following sections.

## Materials and Methods

### Supported Membranes: Materials

NaCl, EDTA, chloroform were purchased from Sigma-Aldrich (Germany) and Hepes, NiCl_2_, CaCl_2_ from Carl Roth GmbH (Germany). 1- Stearoyl-2-oleoyl-*sn*-glycero-3-phosphocholine (SOPC), 1,2-dioleoyl-*sn*-glycero-3-[(N-(5-amino-1-carboxypentyl)-iminodiacetic acid)succinyl] (nickel salt) (DOGS-NTA (Ni^2+^)), obtained from Avanti Polar Lipids (Alabaster, AL, USA), were dissolved in chloroform and stored at −20°C. As solid substrates, AT cut quartz crystals (5 MHz) coated with SiO_2_ (QSX 303, Q-Sense, Gothenburg, Sweden) were used for QCM-D, Si wafers with native oxide (Si-Mat, Landsberg am Lech, Germany) for specular X-ray reflectivity, and glass cover slips (Carl Roth GmbH, Germany) for optical microscopy.

### Supported Membranes: Preparation

If not stated otherwise, all substrates were cleaned by using a modified RCA protocol [42]: the samples were sonicated for 5 min in acetone, ethanol, methanol, and water, then immersed in a solution of H_2_O_2_ (30%)/NH_4_OH (30%)/H_2_O (1∶1∶5 by volume) and sonicated for 5 min at room temperature before soaking them for another 30 min at 60°C. Afterwards, they were intensively rinsed with water, dried at 70°C, and stored in a vacuum chamber. Lipid solutions with different molar ratios of DOGS-NTA in SOPC (0, 1, 2, and 5 mol%) were prepared by mixing appropriate amounts of the stock solutions. After evaporating the solvents under a stream of nitrogen, the samples were placed in a vacuum oven for 24 h in order to remove all traces of solvent. The dry lipid films were then re-suspended in Hepes buffered saline (HBS; 150 mM NaCl, 10 mM Hepes, pH 7.4) at a total concentration of 1 mg/ml. Small unilamellar vesicles (SUVs) were obtained by sonicating lipid solutions with a tip sonicator (Misonix, New York, USA) for 30–60 min, followed by centrifugation in an Eppendorf centrifuge (10 min at 13200×*g*). After the preparation, SUV suspensions were stored at 4°C and diluted to a final concentration of 0.5 mg/ml before use. Supported membranes were prepared by deposition of SUV suspensions on cleaned substrates. After removing excess vesicles by rinsing with buffer, the supported membranes were biofunctionalized by incubating them for 1 h with a solution of Xcad-11 EC1-3 SNAP His12 protein in HBS buffer at a final concentration of ∼20 µg/ml.

### Cloning

Extracellular domain 1–3 (EC1-3) of Xcad-11 [36], [37] was subcloned in pSEMS1-26 m (Covalys, Germany) and a PCR amplified His12-tag was inserted with specific primers (His12_XhoI_fwd: GACTCGAGCAGGAGGAATTAACCATGGCA and His12_NotI_back: GAGCGGCCGCCTGACGGGTAAAGCTCTCCTG). Details of the protein recombination are described in Supporting Information S1. Construction of GAP43-mCherrry, GAP43-GFP [37] and *Xenopus slug* promoter (accession no. AF368040) [34] were described earlier. Specific regions of the *slug* promoter (1–423 bp, 2701–2782 bp, 2797–2961 bp) were fused to GFP and a kind gift of F. Broders. A truncated BMP receptor (tBR) was used as a dominant-negative form and cloned into pSP64. *Xenopus* Frizzled7 (XFz7) DNA was cloned into pCS2+ and a kind gift of H. Steinbeisser.

### *Xenopus* Embryos, Micromanipulation and Explantation

*Xenopus laevis* embryos were obtained by *in vitro* fertilization and staged according to Nieuwkoop and Faber [43]. All animal studies were performed in strict accordance with German Animal Welfare legislation. All protocols and ethical evaluation were approved by the Institutional Animal Welfare Officer (Tierschutzbeauftragter) of the Karlsruhe Institute of Technology, and necessary licenses were obtained from the Regierungspräsidium Karlsruhe, Germany (the regional license granting body; permit numbers: 35-9185.81/G-27/10). Necessary anesthesia was performed under MS-222 and all efforts were made to minimize suffering. Capped mRNAs for injection experiments were synthesized *in vitro* using the mMESSAGE mMACHINE Kit (Ambion; Norwalk, CT, USA). If not indicated otherwise, all injections were performed into the animal hemisphere of one blastomere of a two-cell stage embryo with following amounts: 300 pg tBR RNA, 500 pg XFz7 RNA, 500 pg GAP43-mCherry RNA, 150 pg *slug*-promoter-GFP DNA. The injected embryos were cultivated in 0.1× modified Barth solution holding (MBSH) at 14°C. When the embryos reached blastula stage (stage 8 to 9), the vitelline membrane was manually removed with fine forceps. The presumptive ectodermal sheets (approx. 0.4×0.4 mm, animal caps) were cut from the blastocoel roof of the embryo and placed on supported membranes. Here, the inner blastocoelic surface of the animal caps was facing to the supported membrane. After seeding, the animal caps were cultured in RDX medium [44] supplemented with 1.5 mM Ca^2+^ at 14°C. For more information on animal caps, see Supporting Information S2.

### Quartz Crystal Microbalance with Dissipation (QCM-D)

Quartz crystal microbalance with dissipation (QCM-D) was used to detect the changes in the resonance frequency shift (Δ*f*) and energy dissipation shift (Δ*D*) caused by the membrane deposition and protein binding. When changes in *D* are low (typically <1×10^−6^ per ∼−10 Hz) [38], [45], [46], a decrease in the resonance frequency, Δ*f*, can be translated to an increase in the mass, Δ*m*, via the Sauerbrey equation [47], 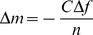. Here, *n* is the overtone number, and *C* is the mass sensitivity constant (which is 17.7 ng cm^−2^ Hz^−1^ for a 5 MHz resonator).

**Figure 2:**
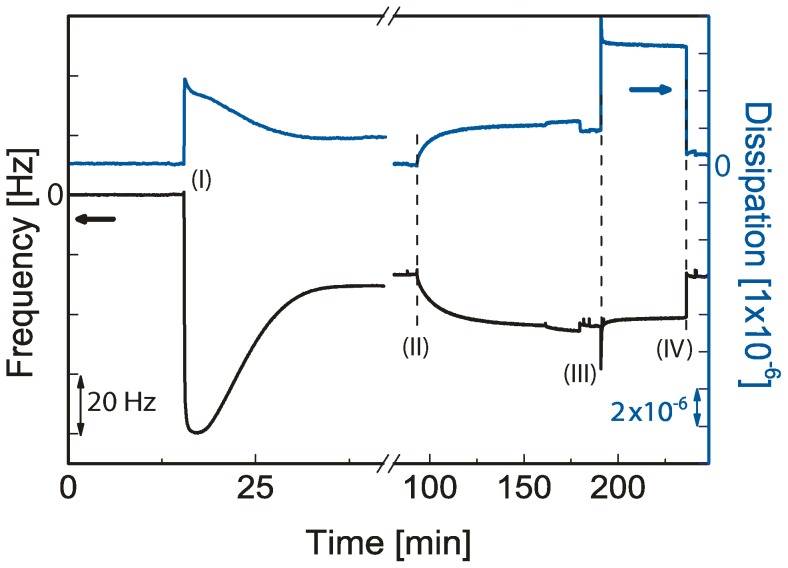
Formation and functionalization of supported membranes. Formation of a supported membrane containing 5 mol% DOGS-NTA (I), specific binding of Xcad-11 EC1-3 SNAP His12 (II) and decoupling of Xcad-11 (through incubation with EDTA-solution (III) and subsequent washing (IV)) monitored by QCM-D. The membrane deposition, protein binding and protein decoupling are detected by changes in the resonance frequency Δ*f* and dissipation Δ*D*.

The QCM-D data were recorded using a D 300 from Q-Sense (Gothenburg, Sweden). To prevent mechanical damage to the crystals, the RCA cleaning method was avoided for the QCM-D samples. Here, SiO_2_-coated QCM-D crystals were cleaned in 10 mM sodium dodecyl sulfate (SDS), rinsed in water, and put in a UV/ozone chamber for 20 min before each measurement. All measurements were recorded at the third overtone (15 MHz) throughout the study. In this paper, all the data were normalized to the fundamental frequency (5 MHz) by dividing the result with the overtone number (3). After establishing a baseline in buffer, suspensions of SOPC vesicles incorporating either 1, 2 or 5 mol% DOGS-NTA were added to the quartz crystal (see step I in Fig. 2). After washing away non-bound vesicles, the change in frequency and dissipation reached a steady state at Δf∼26.0 Hz and ΔD∼0, respectively. This is in very good agreement with previously reported values [48], confirming the formation of a planar lipid bilayer. Protein binding was then achieved by adding an Xcad-11 solution with a concentration of 23 µg/ml.

### High Energy Specular X-ray Reflectivity Measurements

High energy specular X-ray reflectivity measurements were performed at the ID10B beamline at the European Synchrotron Radiation Facility (ESRF, Grenoble, France) by illuminating the samples at 22 keV, which guarantees approx. 40% transmission through 1 cm of water [40], [41]. Prior to the membrane deposition, the cleaned Si wafers were placed into a Teflon chamber with Kapton windows. The momentum transfer perpendicular to the interface is given as a function of the angle of incidence α_i_, 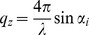. For each measurement point, the reflectivity was corrected for the beam footprint and for the beam intensity with an aid of an in-beam monitor. To minimize the artifacts from radiation damage, we carefully checked the reproducibility of the results by translating the sample position in the direction perpendicular to the beam. The data was fitted by using the Parratt formalism [49] with a genetic minimization algorithm implemented in the Motofit software package [50].

### Optical Microscopy

Live cell images *in vivo* were taken with a microscope observer Z1 (Carl Zeiss AG, Jena, Germany) featured with either a C-ApoChromat 63×/1.2 water-immersion objective or an LD LCI Plan-ApoChromat 25×/0.8 water-immersion objective, a spinning disc unit (Yokogawa Electric Corporation, Tokyo, Japan) and an AxioCam MRm camera (Carl Zeiss AG, Jena, Germany). Reflection interference contrast microscopy (RICM) was performed by using an Axiovert 200 inverted microscope (Carl Zeiss, Göttingen, Germany) equipped with a PlanNeofluar 63×/1.25 Antiflex oil-immersion objective and an Orca ER CCD camera (Hamamatsu Photonics, Herrsching, Germany).

## Results and Discussion

### Functionalization of Supported Membrane with Xcad-11

The binding of Xcad-11 to supported membranes consisting of SOPC/DOGS-NTA mixtures was monitored using QCM-D by recording changes in resonance frequency and dissipation as a function of time (Fig. 2). After formation of a lipid bilayer containing 5 mol% DOGS-NTA (I), the addition of Xcad-11 solution leads to a continuous change in both Δ*f* and Δ*D* (II). After *t*∼70 min and washing off non-specifically adsorbed Xcad-11, a decrease in Δ*f* and an increase in Δ*D* were observed, Δ*f* = 17.3 Hz and Δ*D* = 1.81×10^−6^, respectively. The observed changes in Δ*f* and Δ*D* can be attributed to the increase in the surface mass density by the deposition of a viscoelastic protein layer. The specific binding of Xcad-11 with histidine tag to the chelator head group (Ni^2+^-NTA complex) was verified by dissociating the chelator complex by adding 100 mM EDTA solution (III) followed by the rinsing with buffer (IV). The control experiment performed by adding Xcad-11 on a pure SOPC bilayer did not lead to any adsorption (data not shown).

Since the changes in Δ*D* are in the order of 1×10^−6^ per –10 Hz, changes in the resonance frequency can be translated to the mass densities of Xcad-11 coupled to the membrane using the Sauerbrey equation [47]. It should be noted that it is not possible to directly convert the calculated Δm Xcad-11 [kg/m^−2^] into the protein density [mol/m^−2^], as the coupled mass also contains the mass of hydrating water. Nevertheless, as presented in Table 1, the calculated mass change (Δm Xcad-11) showed a linear increase with the molar fraction of DOGS-NTA. Since SOPC and DOGS-NTA are ideally mixed in our experimental system, the average distance between the DOGS-NTA anchors can be determined as a function of average area per lipid *A*_lipid_ = 65 Å^2^ [51] and molar fraction of DOGS-NTA *χ*_NTA_, 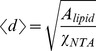. In another set of experiments, we checked the long term stability of the protein binding via NTA-histidine linkers. Similar to our previous experiments with e-GFP and DSRed tetramers with histidine tags [52], QCM-D results confirmed the stability of the protein coupling; the change in Δ*f* was less than 5% over 5 h (data not shown).

**Table 1:** Calculated changes in mass density caused by the coupling of Xcad-11 (Δ*m*) and the average distance between anchor lipids (<*d*>) at different molar fractions of DOGS-NTA anchors (*χ*_NTA_).

NTA lipids in SOPC (*χ*_NTA_)	%	1	2	5
Δm Xcad-11 EC 1–3	ng/cm^2^	45.5	73.2	304.7
<d>	Å	80.6	57.0	36.1

### High Energy Specular X-ray Reflectivity

**Figure 3:**
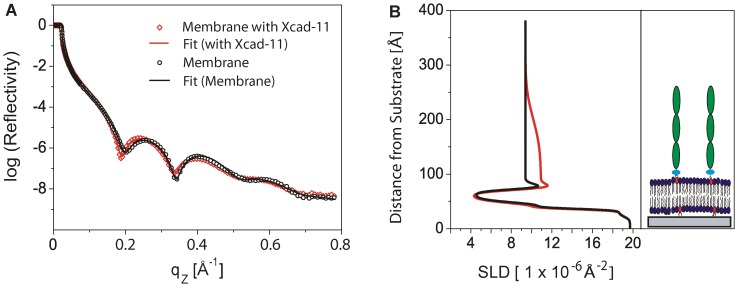
Fine structure of supported membranes probed by X-ray reflectivity. (A) Specular X-ray reflectivity curves of the supported membrane with 5 mol% DOGS-NTA before (black) and after (red) the binding of Xcad-11. The experimental errors are within the symbol size. The solid lines represent the best model fits to the data. (B) The corresponding scattering length density (SLD) profiles demonstrated that the Xcad-11 anchored on the surface at a high density can be treated as a “layer”.

To resolve the fine structures of supported membranes displaying extracellular domains of Xcad-11, we measured specular X-ray reflectivity at a high energy (22 keV) at the solid-liquid interface. Fig. 3A shows the specular X-ray reflectivity curves of the supported membrane with 5 mol% DOGS-NTA before (black circles) and after (red diamonds) the binding of Xcad-11. Both curves exhibit two pronounced minima, indicating the presence of layers with high electron density contrasts. The shift of the positions of the first and second minima towards lower q values in the presence of Xcad-11 indicates an increase in the total thickness by the protein coupling.

The reflectivity of the solid supported membrane (black) was fitted with 5 slabs (outer head groups, hydrocarbon tails, inner head groups, water reservoir, and SiO_2_), while the reflectivity of the membrane functionalized with Xcad-11 was analyzed with a 6 slab model. The best fit results are presented as solid lines in Fig. 3A, and the corresponding scattering length density (SLD) profiles are shown in Fig. 3B. The thickness *d*, SLD, and root mean square (rms) roughness *σ* of each interface are summarized in Table 2 (solid supported membrane) and in Table 3 (membrane functionalized with Xcad-11).

**Table 2:** The best fit parameters for the reflectivity results for the solid supported membrane as presented in Fig. 3.

	Thickness	SLD	Roughness
	[Å]	[10^−6^ Å^−2^]	[Å]
Headgroup(SOPC, DOGS-NTA-Ni)	7.1	12.2	5.0
Alkyl chain	24.8	4.5	3.8
Headgroup(SOPC, DOGS-NTA-Ni)	6.9	12.8	5.1
water	4.0	9.4	3.3
SiO_2_	15.0	18.6	2.4

**Table 3:** The best parameters for the reflectivity results for the membrane functionalized with Xcad-11 as presented in Fig. 3.

	Thickness	SLD	Roughness
	[Å]	[10^−6^ Å^−2^]	[Å]
Xcad-11 EC 1–3	126.0	10.9	44.5
Headgroup(SOPC, DOGS-NTA-Ni)	7.8	13.0	5.4
Alkyl chain	25.5	4.2	4.4
Headgroup(SOPC, DOGS-NTA-Ni)	6.9	12.2	5.8
water	4.0	9.4	3.4
SiO_2_	14.0	18.6	2.4

It should be noted that the thickness, SLD, and roughness of hydrocarbon chains layers remained almost unchanged before and after the protein coupling, which seem comparable to those of fluid lipid membranes [53]–[55]. The other structural parameters, such as the thickness, the roughness and the scattering length densities of the proximal and distal head group regions as well as the thickness of water reservoir between the lipid bilayer and the substrate agree well with those reported in previous accounts [40], [56], [57].

The thickness of Xcad-11 layer with three extracellular domains (EC 1–3) can be estimated to be 126 Å, which seems plausible from the full length of an extended E-cadherin (EC 1–5) molecule (∼220 Å) [58]. The calculated SLD of the protein layer, 10.9×10^−6^ Å^−2^, agrees well with a previous study on another cadherin, C-cadherin [59]. A relatively higher roughness of the Xcad-11/water interface (44.5 Å) than the other interfaces can be attributed to the fact that the Xcad-11 “layer” is composed of a lateral assembly of rod-like cadherin. It should be noted that the reflectivity curves are reproducible up to the instrument resolution even 10 h after the rinsing with HBS buffer, confirming the stability of the membrane-protein coupling.

### Immobilization of Animal Caps on Supported Membranes

**Figure 4:**
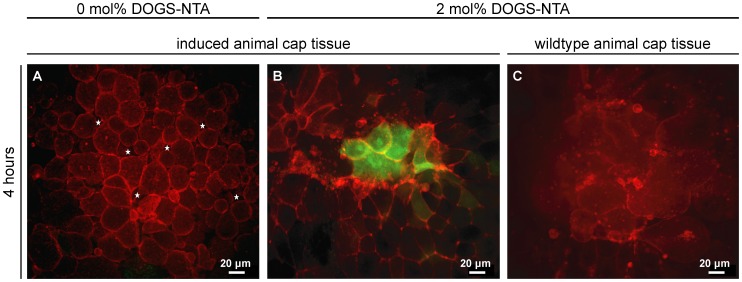
Animal cap tissue incubated on supported membranes: Impact of lateral density of Xcad-11. *Xenopus* embryos were injected in one blastomere of 2-cell stage with 300 pg tBR and 500 pg XFz7 to induce neural crest cell fate (induced animal cap tissue) or left non-induced (wildtype animal cap tissue). Additionally 500 pg GAP43-mCherry was injected as membrane tracer, and 150 pg *slug*-promoter-GFP as read-out for neural crest induction process. Animal cap explants were taken at stage 9 and cultivated on pure SOPC membranes (0 mol% DOGS-NTA, A) and on Xcad-11-functionalized membranes (2 mol% DOGS-NTA, B, C). Animal caps were placed on the surface and imaged after 4 hours of cultivation. Activation of *slug*-promoter-GFP reporter (green) indicates successful neural crest induction. NCC induced tissue started to dissociate and cells lost cell-cell contact (A, gaps are highlighted by asterisks) on pure SOPC membranes. Induced explants on Xcad-11 functionalized surface (B) consist of a cohesive tissue that shows close cell-cell contacts. A clear expression of the *slug* transcription factor was detected by the positive GFP signal. Wildtype animal cap tissue disintegrated and started dying (C) on functionalized membranes.

Following the full characterization of supported membranes functionalized with Xcad-11, the potential of the planar membranes for the long-term cultivation of cohesive tissue sheets was examined. Using confocal fluorescence microscopy, we monitored the cell behavior, cell survival and the induction of neural crest cells in animal caps over 4 h (see Table 4 for an overview of all performed experiments). *Xenopus* embryos were injected in one blastomere of two-cell stage with 300 pg tBR and 500 pg XFz7 to induce neural crest cell fate (“induced” animal cap tissue; for a detailed proof of successful induction performed by Real Time PCR, see Supporting Information S3) or were left non-induced (“wildtype” animal cap tissue). To visualize cell membranes, 500 pg GAP43-mCherry was additionally injected as cell membrane tracer. As presented in Fig. 4, the induced animal cap incubated on a pure SOPC membrane containing no Xcad-11 showed no sign of adhesion (i.e. no formation of filopodia, see also Table 4; in Supporting Information S4 we demonstrate, by showing a z-stack, that we are indeed imaging the substrate/explants contact region, i.e. the bottommost cell layer of the animal cap) and eventually lost cohesion between cells in the tissue sheet, which was visible by the formation of gaps between cells. This results in a spherical cell morphology, indicating cell death (Fig. 4A; Supporting Information S5). Moreover, we observed that induced animal caps did not survive on supported membranes that display RGD peptides specific to integrin receptors at the average distance of <*d*>∼5.7 nm. Here, individual cells showed strong adhesion to the surface, but the cells lost their connective structural integrity and died after 2 h (Table 4). These findings suggest that functionalization of supported membranes with inadequate adhesion motifs disturbs tissue cohesiveness most likely by altering mechanical forces or intercellular communication within the animal cap. Similar effects of cohesiveness were discussed in a previous report studying cell polarization at tissue boundaries. Cell polarization was only observed when pieces of cohesive tissues but not single cells of different origin were combined [21].

**Table 4:** Overview on the behavior of induced and wildtype animal caps on differently modified surfaces (Xcad and Ecad functionalized lipid membranes (*d*∼<5.7 nm>, non-functionalized lipid membrane and RGD functionalized lipid membrane (*d*∼<5.7 nm>).

	Induced tissue		Wildtype tissue	
*Incubation time*	*0 h*	*4 h*	*0 h*	*4 h*
**Xcad-11**	Adhesion, cohesive tissue, filopodia		No adhesion, tissue dissociation, cell death	
*Nr. of samples (Xcad-11)*	*13/18*	*13/18*	*11/20*	*5/20*
**Ecad**	No adhesion, tissue dissociation, cell death		Adhesion, cohesive tissue, filopodia	
*Nr. of samples (Ecad)*	*3/15*	*3/15*	*15/18*	*4/18*
**SOPC**	No adhesion, tissue dissociation, cell death		No adhesion, tissue dissociation, cell death	
*Nr. of samples (SOPC)*	*2/10*	*2/10*	*2/10*	*0/10*
**RGD**	Strong initial adhesion, loss of cohesiveness after 2 h		Adhesion	
*Nr. of samples (RGD)*	*6/9*	*3/9*	*2/5*	*3/5*

The numbers refer to the fraction of animal caps adhering to the surface after 0 and 4 h, out of the total number of explants examined in each case.

We succeeded in the stable cultivation of induced animal caps on supported membranes displaying Xcad-11 (Fig. 4B), which can be attributed to the fact that a switch in the cadherin profile accompanies the induction of neural crests. While non-induced (wildtype) animal caps contain the maternally provided C- and XB-cadherin [60]–[62], neural crest cells (NCC) express Xcad-11 and N-cadherin instead [36], [63]. Xcad-11 does not only mediate the cell-cell adhesion, like other classical cadherins, but also guides the formation of cell protrusions [37]. This finding suggests that a successful induction of NCC is necessary for the binding of animal caps to the membrane functionalized with Xcad-11. In contrast, non-induced, wildtype animal cap tissues were disintegrated and started dying (Fig. 4C) on Xcad-11 functionalized membranes. In order to verify that interactions between the induced tissue sheets and membrane are specifically mediated via homophilic Xcad-11 binding, animal cap tissues were cultured on membranes displaying E-cadherin EC1-5 SNAP His12 at a lateral distance of <*d*>∼5.7 nm. Since wildtype animal caps differentiate into atypical epidermis and express E-cadherin [64], wildtype tissue sheets showed a strong adhesion and formed cell protrusions (Supporting Information S6D–F). In contrast, NCC induced animal caps that express Xcad-11 [65] were unable to adhere (Supporting Information S6A–C).

The change into neural crest cell fate in induced animal caps can be further monitored by the activation of the NCC marker gene *slug* [34], [66]. The transcription factor *slug* is one of the earliest specifiers of neural crest cell fate. To confirm the induction of NCC, we injected a reporter fusion construct consisting of the minimal *slug* promoter and GFP (Fig. 4). As presented in Fig. 4B, the activation of the *slug* reporter could be detected by GFP signals on supported membranes displaying Xcad-11. Since *slug* is expressed only at early stages of NCC determination, the obtained result confirmed the successful induction of NCC in explanted *Xenopus* animal cap cells on supported membranes functionalized with Xcad-11.

**Figure 5:**
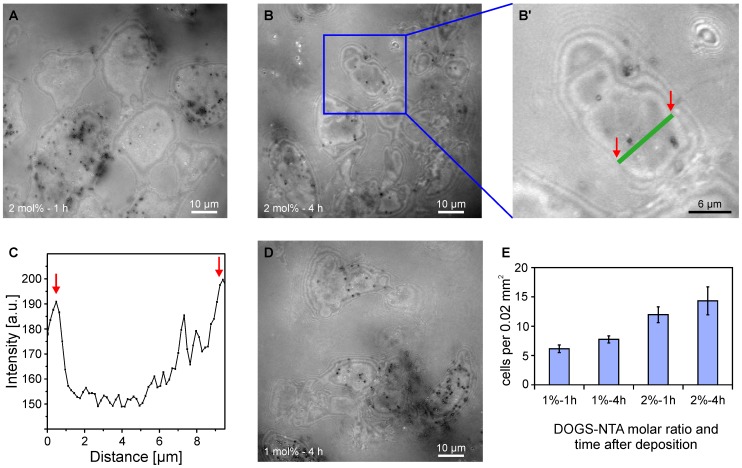
Time evolution of contacts between induced animal cap tissues and supported membranes displaying Xcad-11. With the aid of microinterferometry (RICM), the region of tight cell-surface contacts can be identified as the darker patches (A, B, D). It should be noted that not all the cells in the tissue sheet adhered to the surface, although the animal cap retained a connective structure. (A, B) Animal caps on membranes containing 2 mol% DOGS-NTA (<*d*>∼5.7 nm) after 1 h and 4 h incubation, respectively. Panel (B’) shows the enlargement of the blue rectangle in panel B and panel (C) shows the intensity profile along a green line in the panel B’, reflecting the accumulation of adhesion patches towards the cell center. (D) Animal cap on a membrane containing 1 mol% DOGS-NTA (<*d*>∼8.1 nm) after 4 h. (E) The number density of cells in tissue sheets with adhesion patches larger than 30 µm^2^ within an area of 20000 µm^2^ showed a clear increase with increasing molar ratio of DOGS-NTA and time, suggesting that the adhesion of animal caps on membranes displaying Xcad-11 is specifically mediated by Xcad-11 and develops as a function of time. At least 3 explants per condition were used to make the histograms. Small black spots in A, B, B’ and D are pigment granules inside ectoderm cells and therefore negligible



Fig. 5A represents the RICM image of an animal cap that was in contact with a supported membrane displaying Xcad-11 molecules at an average distance <*d*>∼5.7 nm (corresponding to 2 mol% DOGS-NTA) for 1 h. As the interference of linearly polarized light reflected from the substrate surface and the cell surface becomes destructive at low cell-surface distances (typically below 10 nm), the regions of tight cell-substrate contact can be identified as dark patches [67]–[69]. Here, the intensity of the interference signal *I* reflects the local distance between the cell and the substrate by:

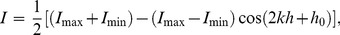
(1)and the interference “fringes” surrounding the patches are originated from the higher order minima. This makes the RICM technique highly unique for the label-free, live tissue imaging that enables one to clearly identify the region of tight tissue-membrane contacts, which cannot be assessed by confocal microscopy. Moreover, the fluctuation of pixel intensity, i.e. the probability function of the cell-surface distance *P*(*h*), sensitively reflects the effective interaction potential *V*(*h*), which can be described within the framework of an inverse work function theorem: 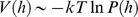 [15], [70].

**Figure 6:**
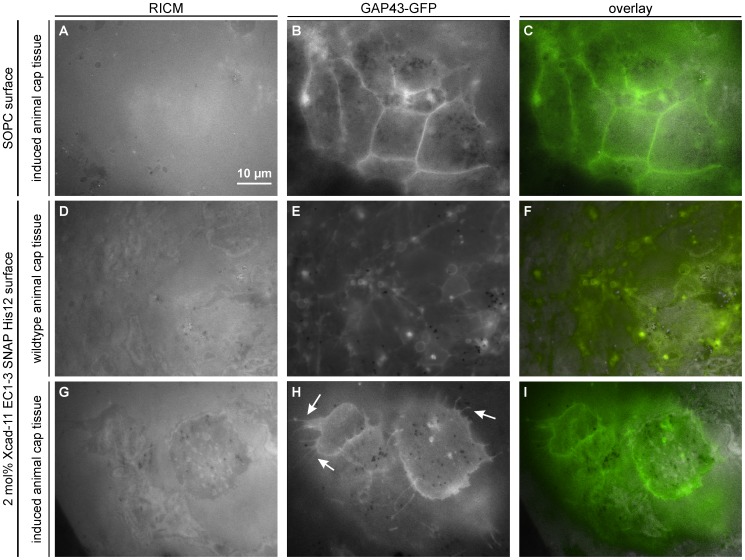
Induced and wildtype animal cap tissues on supported membranes with/without Xcad-11 (t = 4 h). The animal cap cells were labeled with GAP43-GFP for visualization of the membrane. (A–C) Induced tissue did not adhere on non-functionalized SOPC membranes, showing no adhesion patch (A) and no filopodia (B). Image B is take at z∼2 µm above image A. (D–F) Wildtype tissue cultivated on a supported membrane functionalized with Xcad-11 EC1–3 at <*d*>∼5.7 nm. Although some cells in tissue sheets adhered to the supported membrane, the intensity fluctuations near the cell center are still remarkable (D). The tissue exhibited membrane budding (E, F), which is characteristic for disintegrated tissues. Images D and E are taken at the same z position. (G–I) NCC induced animal cap on a Xcad-11 functionalized membrane (<*d*>∼5.7 nm). As presented in panel (G), the fluctuation of cell-surface distance was strongly damped, compared to wildtype tissues on the same surface (D). The formation of filopodia and lamellipodia characteristic for neural crest cells (H and I, indicated by arrows) demonstrate that induced tissues remain viable and can properly shape the cells on membranes functionalized with Xcad-11. Images G and H are taken at the same z position.

As presented in Fig. 5, only a few portions of cells in the animal cap tissues were adhered to the surface, although they retained a connective tissue structure. At the earlier stage of the cultivation (Fig. 5A, *t* = 1 h), adhesion patches were mainly found near the cell periphery, which seems plausible from the initial accumulation of Xcad-11 in cell protrusions [71]. When the animal caps were further incubated (Fig. 5B, *t* = 4 h), the area of strong contact became larger, implying the coalescing of Xcad-11 pairs towards the middle of the cells [15], [72], [73]. We analyzed this quantitatively by determining the ratio of the strong adhesion area (here defined as the regions darker than 
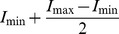
) relative to the area of the whole microscope image. We found an increase from 4.9% ±1.8 strong adhesion area at t = 0 h to 5.8% ±2.5 at t = 4 h (n = 15 explants). The increase in the contact area as well as the accumulation of adhesion patches towards the cell center can be identified by the pixel intensity *I* along a randomly selected contour (green line in Fig. 5C). In contrast, no adhesion patches were observed on pure phospholipid membranes (Fig. 6) or on membranes functionalized with E-cadherin (Supporting Information S6A–C), confirming the specific immobilization of tissue sheets via Xcad-11. Moreover, the tissue sheets were not able to adhere to the Xcad-11 functionalized membranes, when Xcad-11 synthesis was blocked by the injection of antisense Xcad-11 morpholinos (Supporting Information S6G–I).

It should be noted that the quantitative reconstruction of the height profile from the pixel intensity was practically not possible in this study because: (i) the intensity from the first maximum (*I*_max_) and minimum (*I*_min_) can hardly be discriminated from the background by the intensity threshold algorithm, and (ii) the average height *h*_0_ is a product of the refractive indices of all the media (including several cell layers in animal caps) that are not quantitatively determined. Nevertheless, despite of the lack of quantitative information of the cell-surface distance, the flattening of the cell membrane between the first order maxima (*x* = 0.7 µm and 9.2 µm, indicated with red arrows in Fig. 5C) suggests the suppression of the thermal fluctuation of the membrane in the area of tight adhesion [15], [70]. Fig. 5D represents the RICM image of the animal cap incubated on the membrane with <*d*>∼8.1 nm (1 mol% DOGS-NTA) after 4 h. Here, the area of tight adhesion is much smaller and the intensity fluctuation near the cell center is more prominent than those displaying Xcad-11 at <*d*>∼5.7 nm (Fig. 5B), which seems consistent with the previous reports on synthetic lipid vesicles [15]. Our experimental finding suggests that the adhesion of animal cap cells is highly sensitive to the distance between Xcad-11 molecules in nm accuracy. As presented in Fig. 5E, the number of cells with adhesion patches larger than 30 µm^2^ per view area of 2×10^4^ µm^2^ increases in accordance with an increase in the lateral density and thus a decrease in the average distance between Xcad-11, implying trans-interaction of Xcad-11 on the supported membrane with the endogenous Xcad-11 on the cell surface [59], [74]. Moreover, the increase in the number of adherent cells from 1 h to 4 h verifies the compatibility of supported membranes to sustain the stable immobilization of tissue sheets. Further increase in the surface density of Xcad-11 (<*d*>∼3.6 nm, corresponding to 5 mol% DOGS-NTA) did not result in any improvement in immobilization of animal cap tissue sheets, which can be attributed to steric hindrance. Thus, we concluded that the supported membranes displaying Xcad-11 at <*d*>∼5.7 nm can serve as compatible and sustainable surfaces for the immobilization of animal caps without disrupting intercellular connections in tissue sheets.

In order to further verify the connectivity of cells in each animal cap, we imaged the same animal cap tissue sheets using RICM and fluorescence microscopy (Fig. 6). The animal cap cells were labeled with GAP43-GFP for visualization of the membrane. On a pure phospholipid membrane, the RICM image showed no sign of tight adhesion (Fig. 6A) and the corresponding fluorescence image (Fig. 6B) implied that cells do not extend any protrusion. Further incubation leads to disintegration of cohesive tissues, resulting in the cell death. When untreated wildtype animal caps were cultivated on membranes exposing Xcad-11 at a lateral distance of <*d*>∼5.7 nm, the RICM image (Fig. 6D) suggested the adhesion of some cells in tissue sheets, but the intensity profile still exhibited remarkable fluctuations near the cell center. The fluorescence image of the same tissue sample (Fig. 6E) showed that the cells were not able to form filopodia and lamellipodia. Indeed, we observed the formation of many membrane buds (E, F), which are characteristic for disintegrated tissues. This suggests the loss of structural integrity of cell membranes and thus the cell death (Fig. 6E), which is in agreement with Fig. 4C.

The RICM image of the induced animal caps deposited on the membranes functionalized with Xcad-11 (<*d*>∼5.7 nm) showed a clear sign of adhesion, where the fluctuation of cell-surface distance was strongly damped (Fig. 6G). In the corresponding fluorescence image (Fig. 6H) we observed the formation of filopodia and lamellipodia (highlighted with white arrows), which is characteristic for neural crest cells [29]. It should be noted that the suppression of the fluctuation of cell-substrate distance and the formation of filopodia and lamellipodia were observed only for induced tissues on membranes functionalized with Xcad-11. We also observed that the number of filopodia per mm^2^ in the induced tissue increased from an average of 3.9 at t = 0 h up to 6.5 after 4 h. This finding confirms that induced tissue sheets adhere, remain viable, and properly shape the cells on supported membranes functionalized with Xcad-11.

## Conclusions

We demonstrated the immobilization of pluripotent animal caps isolated from embryos of *Xenopus laevis* on supported membranes quantitatively functionalized with recombinant Xcad-11 without disrupting the intercellular connections in tissue sheets. After the full characterization by the combination of QCM-D and high energy X-ray reflectivity, we cultivated planar animal cap tissue sheets on the supported membranes. The adhesion behavior of animal cap tissue sheets induced to neural crest cell (NCC) strongly depended on the lateral density of Xcad-11, and the formation of filopodia and lamellipodia in the cohesive tissue verified the viability of the animal caps over several hours. We confirmed the specific regulation of NCC induction by cultivating different animal cap tissues (wildtype tissues and tissues treated with Cad-11 morpholino oligonucleotide) on membranes functionalized with Xcad-11, on pure phospholipid membranes, as well as on membranes functionalized with recombinant E-cadherin. After the optimization of the surface density of Xcad-11 (<*d*>∼5.7 nm), we achieved the expression of the transcription factor *slug* in induced tissue samples. Thus, the obtained results suggest that supported membranes functionalized with Xcad-11 can serve as compatible and sustainable substrates for long-term culture of cohesive pluripotent tissue sheets from *Xenopus laevis*.

## Supporting Information

Supporting Information S1 **Expression and purification of proteins.**
(DOC)

Supporting Information S2 **Explanation for Animal caps of Xenopus laevis.**
(DOC)

Supporting Information S3 **Successful induction of neural crest tissue fate in animal caps by injection of tBR and Fz7.**
(DOC)

Supporting Information S4 **The tissue – membrane interface was identified by recording z-stacks of the animal caps.**
(DOC)

Supporting Information S5 **Loss of tissue cohesion.**
(DOC)

Supporting Information S6 **Specificity of tissue – membrane interaction.**
(DOC)
