# Supplementary material for: Cell Differentiation of Pluripotent Tissue Sheets Immobilized on Supported Membranes Displaying Cadherin-11
Source: PLoS One. 2013 Feb 12;8(2):e54749. doi: 10.1371/journal.pone.0054749 (PMC3570561; doi:10.1371/journal.pone.0054749)
Supplement: Supporting Information S1 — Expression and purification of proteins. (DOC) [file pone.0054749.s001.doc]

Supporting Information S1: Expression and purification of proteins

Xcadherin-11 EC1-3 SNAP His12 protein was used for biofunctionalization of supported membranes. The protein was produced in SW-1353 cells cultured in DMEM at 7% CO_2_ and 37°C. Comparison with expression in HEK293 (human embryonic kidney cells) and MC3T3 (mouse embryonic calvarium cells) displayed a three times higher expression of Xcad-11 in SW-1353. Cells were stably transfected by using FuGENE 6 transfection reagent (Roche, Mannheim, Germany). Supernatant of cells was harvested every two days, centrifuged at 4000 rpm and stored at 4°C. 500 ml of supernatant were filtered (0.2 µm), concentrated by VivaCell 250 (MWCO: 30000 Da; Sartorius, Göttingen, Germany) and loaded to an FPLC-IMAC to purify the His-tagged Xcad-11 from the crude extract by Ni^2+^-NTA chromatography. Fractions of IMAC protein purification were separated by SDS-PAGE. Fractions with purified Xcad-11 protein were identified on Western blots, combined, and stored at -80°C after addition of 1 mM DTT. Western blots were carried out as described [1]. Rabbit polyclonal anti-SNAP antibody was obtained from Thermo Fisher Scientific (1:500; Schwerte, Germany) and secondary antibody goat anti rabbit peroxidase conjugated from Dianova (1:10000; Hamburg, Germany). Silver staining of SDS gel according to Jensen et al. [2] was used as loading control. Xcad-11 EC1-3 SNAP His12 was also identified on MALDI (data not shown).

**References:**

1. Unterseher F, Hefele JA, Giehl K, De Robertis EM, Wedlich D, et al. (2004) Paraxial protocadherin coordinates cell polarity during convergent extension via Rho A and JNK. EMBO J 23: 3259-3269.

2. Jensen ON, Wilm M, Shevchenko A, Mann M (1999) Sample preparation methods for mass spectrometric peptide mapping directly from 2-DE gels. Methods in molecular biology 112: 513-530.
