# Supplementary material for: Cell Differentiation of Pluripotent Tissue Sheets Immobilized on Supported Membranes Displaying Cadherin-11
Source: PLoS One. 2013 Feb 12;8(2):e54749. doi: 10.1371/journal.pone.0054749 (PMC3570561; doi:10.1371/journal.pone.0054749)
Supplement: Supporting Information S2 — Explanation for Animal caps of Xenopus laevis. (DOC) [file pone.0054749.s002.doc]

Supporting Information S2:

Explantation of animal caps of *Xenopus laevis*

The *Xenopus laevis* embryo is a well studied system for investigation in developmental biology. Basic steps in tissue induction and morphogenetic movements have been made in this organism. The animal cap assay is one of the key assay systems to investigate inductive signals. The animal cap is the blastocoel roof of a blastula embryo. The single cell oocyte starts dividing after fertilization. After several divisions a compact morula is formed. For the formation of the blastula a cavity is formed at the animal half of the embryo. The cells located there have an ectodermal/neuroectodermal differentiation potential. Grunz et al. [1] showed that an explant of these cells in culture will form an atypical form of epidermis and will roll up to form an ectodermal sphere. For a culture as a sheet after explantation (Fig. S2) a fixation of the explant by a functionalized substrate is necessary to prevent the rolling up. Interestingly, these cells still have a pluripotent character similar to embryonal stem cells and therefore, can form a variety of cell types depending on the growth factors applied in the culture medium or by injection in the early embryo (1- until 4- cell stage). This makes the animal caps a valuable to tool to study cell differentiation. An injection of inhibitors of the BMP signaling pathway (like chordin, noggin or truncated receptors) leads to the formation of neuroectodermal tissue as it can be demonstrated by the expression of neural marker genes. For induction of neural crest a network of different signaling pathways was described: Wnt activation, BMP inhibition in combination with activation of FGF and/or retinoic acid pathway are necessary. In vitro a combination of Wnt activation and BMP inhibition has been shown to be sufficient.


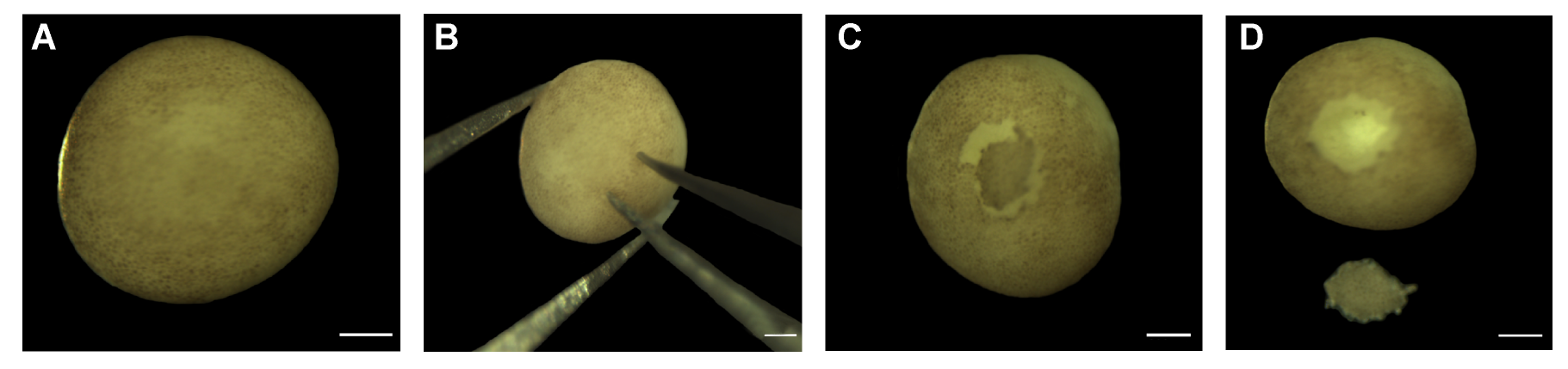


Fig. S2: Procedure of Animal cap explantation: (A) Animal view of a *Xenopus* gastrula stage embryo. (B) At the animal side the blastocoel roof can be explanted by fine forceps. (C) Loosened animal cap. (D) Removed animal cap (below). Scale bar: 200 µm

**Reference:**

1. Grunz H, Multier-Lajous AM, Herbst R, Arkenberg G (1975) The Differentiation of Isolated Amphibian Ectoderm with or without Treatment with an Inductor. Wilhelm Roux's Archives227: 277-283.
