# Supplementary material for: Cell Differentiation of Pluripotent Tissue Sheets Immobilized on Supported Membranes Displaying Cadherin-11
Source: PLoS One. 2013 Feb 12;8(2):e54749. doi: 10.1371/journal.pone.0054749 (PMC3570561; doi:10.1371/journal.pone.0054749)
Supplement: Supporting Information S3 — Successful induction of neural crest tissue fate in animal caps by injection of tBR and Fz7. (DOC) [file pone.0054749.s003.doc]

Supporting Information S3: Successful induction of neural crest tissue fate in animal caps by injection of tBR and Fz7.


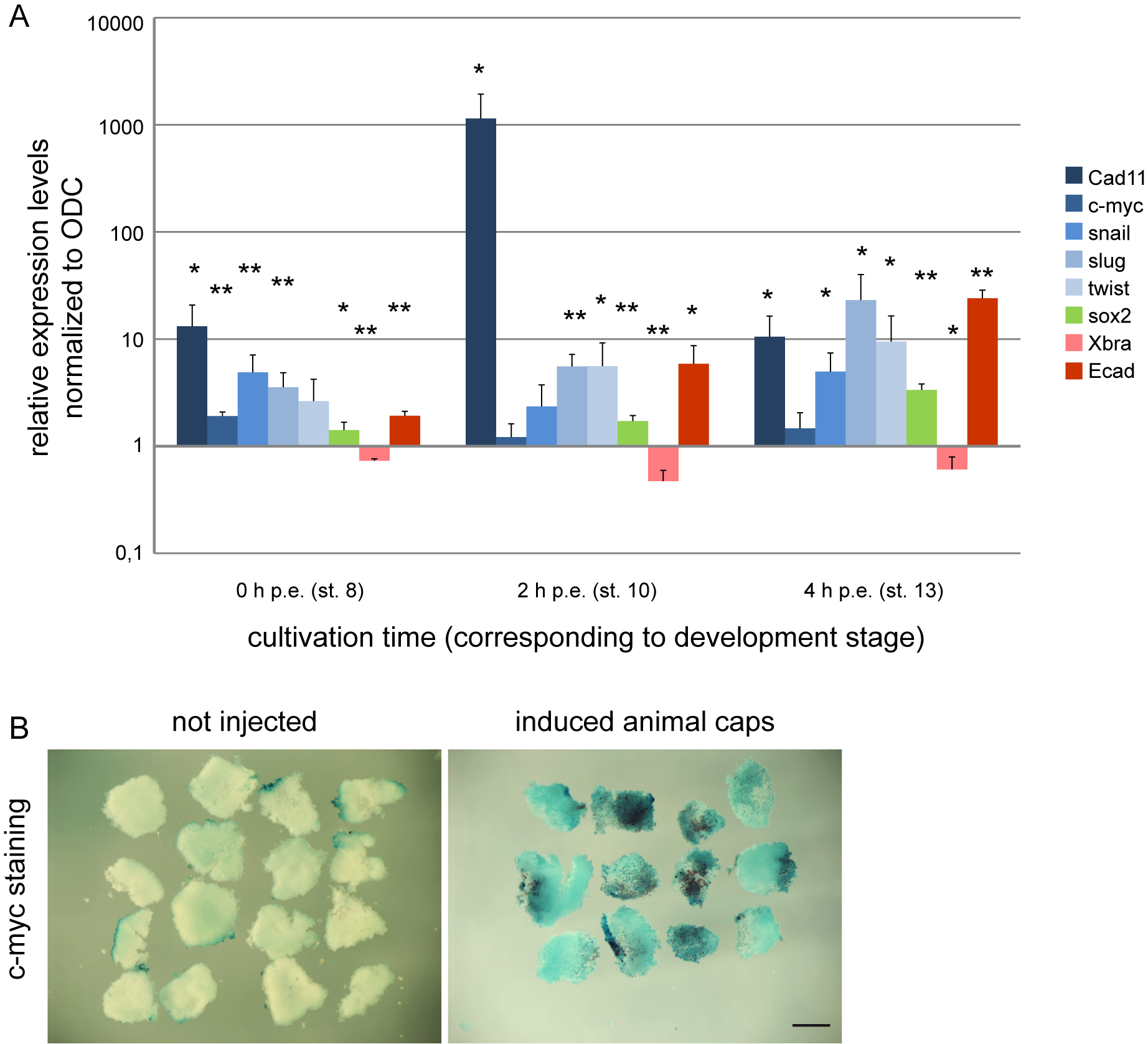


(A) Real Time PCR analysis shows that different neural crest markers (blue) are induced by injection of 300 pg *tBR* and 500 pg *Fz7*. Total RNA was extracted from ten animal cap explants and reverse transcribed. Real-time PCR was performed using iQ SYBR Green Supermix on an iCycler (BioRad, Hercules, CA, USA). Expression levels were calculated relative to *ornithine decarboxylase* (*ODC*) and normalized. Relative expression of the induced animal caps was compared to uninjected wildtype animal caps of stage 8. Results of at least four independent experiments were averaged, and statistical significance was calculated using Student's *t*-test (* p < 0.2, ** p < 0.1). Early markers (*Cad-11*, *c-myc*) are mainly increased already directly after animal cap dissection (st. 8) and in early stages of culture (st. 10) and decrease thereafter, later neural crest markers (*snail*, *slug*, *twist*) are increasing during incubation time. Neural induction as shown by expression of *sox2* (green) is moderate. Mesodermal markers are repressed as seen by *Xbra* (light red) expression. Ectodermal markers (*Ecad*, red) start to be enhanced after longer culture, but not in early stages of animal cap. (B) *In situ* hybridization of animal caps shows increase in expression of *c-myc* in explants injected with *tBR* and *Fz7* directly after explantation by staining with BCIP (blue). Injection is marked by Rose Gal staining (red). Scale bar: 100 µm

Acknowledgement: We gratefully thank Marisa Braun for performing the animal cap *in situ* hybridization.

Sequences of the real-time primers:

Xcad-11_fwd 5' TCG GAT ACT GTG GTC GGA AG 3'

Xcad-11_rev 5' CAT CCT CTG GGT TGA TGC TG 3'

c-myc_fwd 5' TTC CCA TTC ACC AAC ACA ACT A 3'

c-myc_rev 5' GAC CCG GAT GTT GCT TTC TA 3'

Xsnail1_fwd 5' GGC ACC AGT TAT TGC CTT TC 3'

Xsnail1_rev 5' TGT TGT TCC ATC CAC CTG TC 3'

Xslug_fwd 5' ACC TGC AGA CCC ATT CTG AT 3'

Xslug_rev 5' CAC AGC AAC CAG ATT CCT CAT 3'

twist_fwd 5' CTC AGT GAA GCG CAA CAA GA 3'

twist_rev 5' CTC TGA CGC TCC CTG ACA TT 3'

sox2_fwd 5' GAG GAT GGA CAC TTA TGC CCA C 3'

sox2_rev 5' GGA CAT GCT GTA GGT AGG CGA 3'

Xbra_fwd 5' TTC AGC CTG TCT GTC AAT GC 3'

Xbra_rev 5' TGA GAC ACT GGT GTG ATG GC 3'

Ecad_fwd 5' CGA CCT TTG GAC AGA GAA GC 3'

Ecad_rev 5' GCA CAG AGC CTT CAA AGA CC 3'

ODC_fwd_57 5' CAT TGC AGA GCC TGG GAG ATA 3'

ODC_rev_57 5' TCC ACT TTG CTC ATT CAC CAT AAC 3'

ODC_fwd_62 5' GCC ATT GTG AAG ACT CTC TCC ATT C 3'

ODC_rev_62 5' TTC GGG TGA TTC CTT GCC AC 3'
