# Supplementary material for: Cell Differentiation of Pluripotent Tissue Sheets Immobilized on Supported Membranes Displaying Cadherin-11
Source: PLoS One. 2013 Feb 12;8(2):e54749. doi: 10.1371/journal.pone.0054749 (PMC3570561; doi:10.1371/journal.pone.0054749)
Supplement: Supporting Information S4 — The tissue – membrane interface was identified by recording z-stacks of the animal caps. (DOC) [file pone.0054749.s004.doc]

Supporting Information S4: The tissue – membrane interface was identified by recording z-stacks of the animal caps


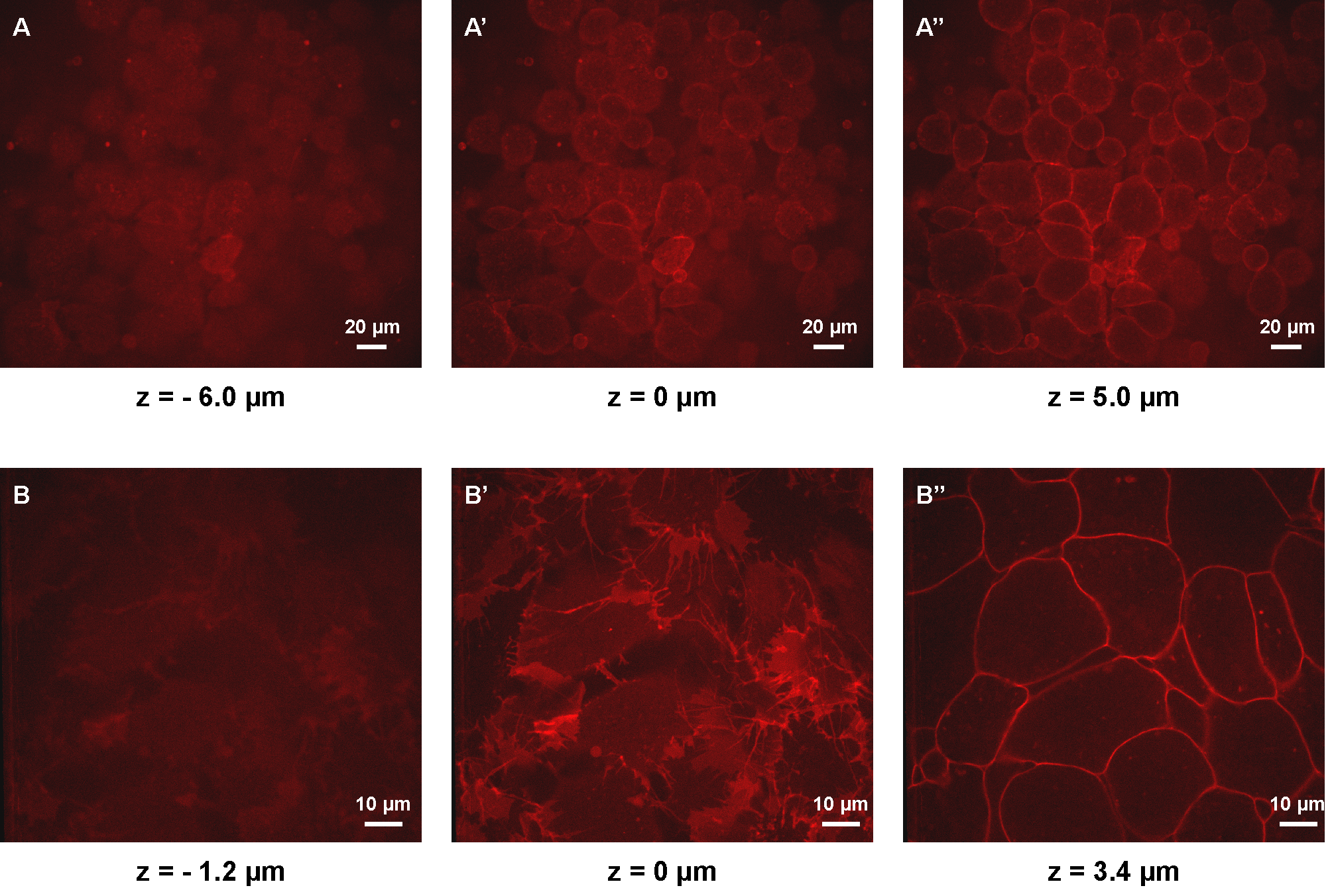


(A-A’’) Tissue-membrane interface as a function of z-position for the induced animal cap shown in Figure 4A after 4 h of culture on a non-functionalized SOPC membrane. The image shown in Figure 4A was taken 5.0 µm above the tissue-membrane interface.

(B-B’’) Tissue-membrane interface as a function of z-position for another induced animal cap at higher magnification. The contact area at z = 0 µm can be easily identified, thus allowing us to confidently state that we are imaging the bottom cell layer of the animal cap.

The cell membranes were stained with GAP43-mCherry.
