# Supplementary material for: Cell Differentiation of Pluripotent Tissue Sheets Immobilized on Supported Membranes Displaying Cadherin-11
Source: PLoS One. 2013 Feb 12;8(2):e54749. doi: 10.1371/journal.pone.0054749 (PMC3570561; doi:10.1371/journal.pone.0054749)
Supplement: Supporting Information S5 — Loss of tissue cohesion. (DOC) [file pone.0054749.s005.doc]

Supporting Information S5: Loss of tissue cohesion.


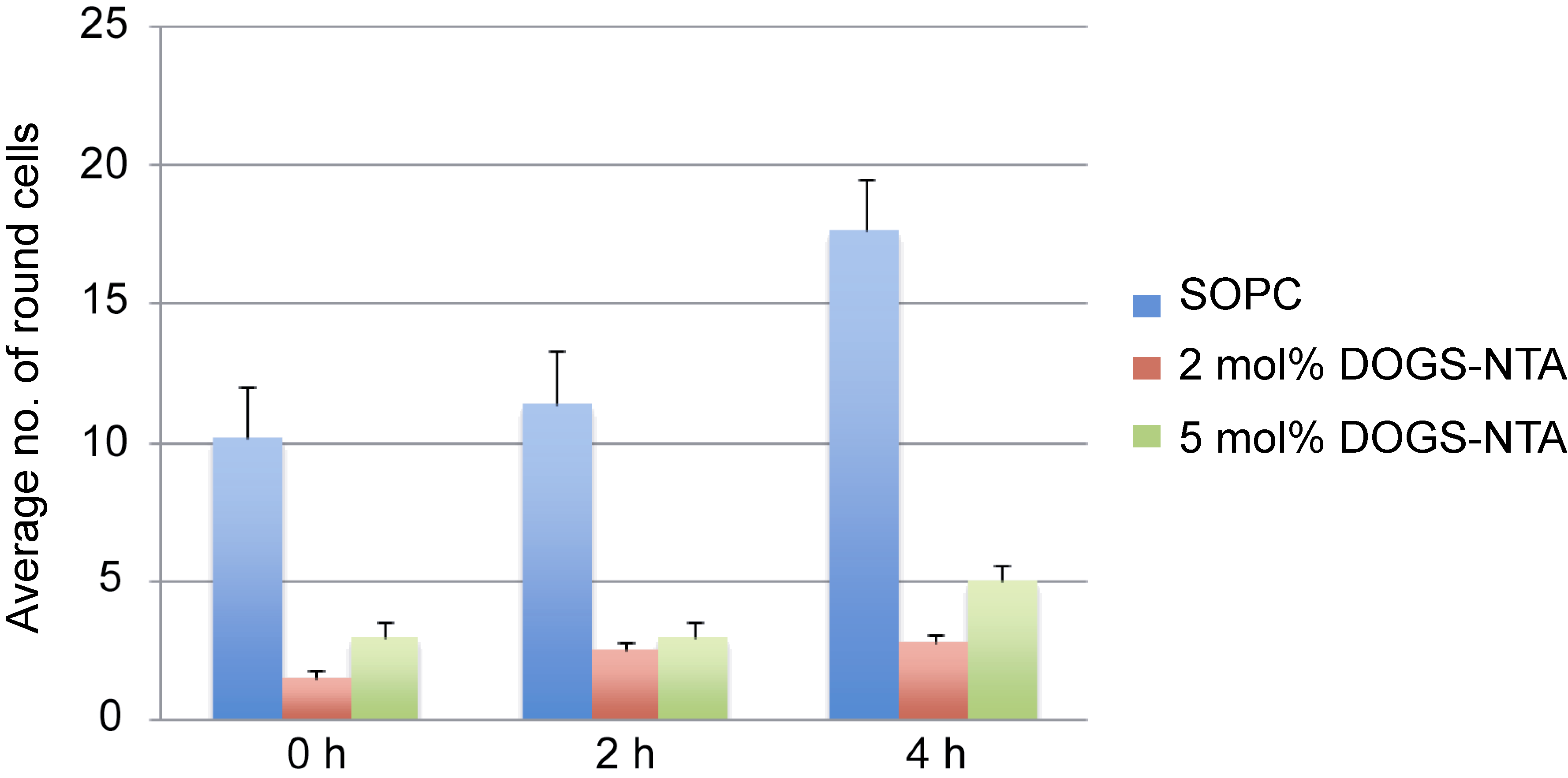


Average number of round cells in 0.08 mm^2^. Explants cultured on pure SOPC membranes showed an increased number of rounded cells compared to culture on Xcad-11 functionalized membranes. Membranes with 5 mol% Xcad-11 resulted in a slightly higher number of round cells than membranes with 2 mol%, most likely due to steric hindrance interfering with adhesion. (Number of explants analyzed for each surface at the SOPC surface: 11, 7, 3 at 0 h, 2 h and 4 h respectively: SOPC: n=11, 7, 3; 2mol%: n=6, 4, 4; 5mol%: n=5, 6, 3)
