# Supplementary material for: Cell Differentiation of Pluripotent Tissue Sheets Immobilized on Supported Membranes Displaying Cadherin-11
Source: PLoS One. 2013 Feb 12;8(2):e54749. doi: 10.1371/journal.pone.0054749 (PMC3570561; doi:10.1371/journal.pone.0054749)
Supplement: Supporting Information S6 — Specificity of tissue – membrane interaction. (DOC) [file pone.0054749.s006.doc]

Supporting Information S6: Specificity of tissue – membrane interaction


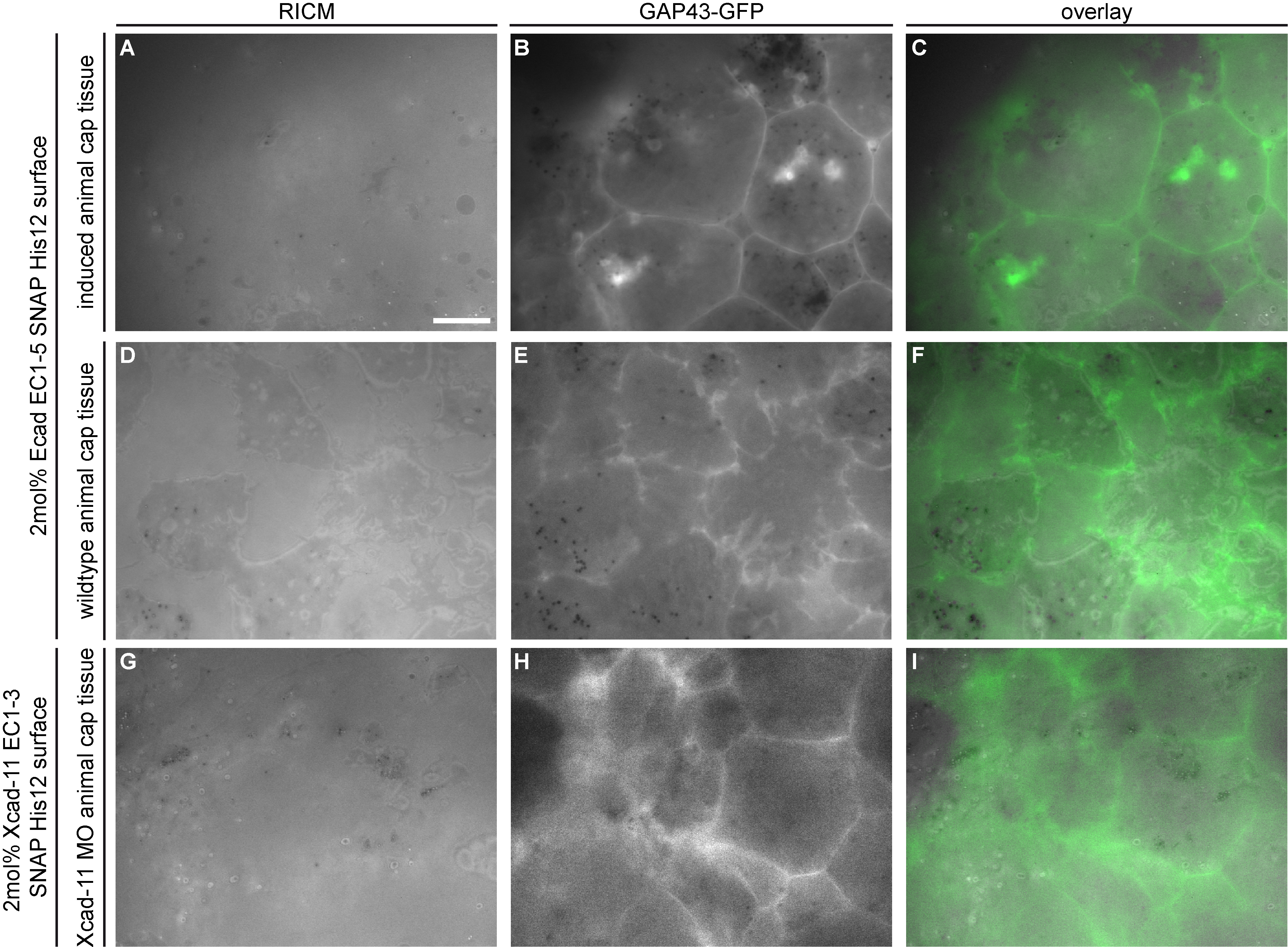


Cultivation of animal caps on Ecad EC1-5 SNAP His12 functionalized membranes (**A-F**) and on Xcad-11 EC1-3 SNAP His12 functionalized membranes (**G-I**). While NC induced animal caps (**A-C**) display expression of Xcad-11 and therefore, show no adhesion to Ecad (resulting in no adhesion patches formed and no formation of cell protrusions), wildtype animal caps (**D-F**) with expression of Ecad show clear adhesion patches and the formation of filopodia. This indicates the specificity of the membrane-tissue interaction observed. (**G-I**) Animal caps with reduced expression of Xcad-11 due to treatment with Morpholino oligonucleotides that repress the translation of specific mRNA are unable to adhere to Xcad-11 SNAP His12 functionalized membranes. All images were taken after 4 hours cultivation.
